# Supplementary material for: Comprehensive proteolytic profiling of Aedes aegypti mosquito midgut extracts: Unraveling the blood meal protein digestion system
Source: PLoS Negl Trop Dis. 2025 Feb 6;19(2):e0012555. doi: 10.1371/journal.pntd.0012555 (PMC11838913; doi:10.1371/journal.pntd.0012555)
Supplement: Attachment — Submitted filename: Response to Reviewers.pdf [file pntd.0012555.s003.pdf]

December 22, 2024

Dear PLoS NTD Editors,

We have updated and changed the manuscript: "**Comprehensive proteolytic profiling of *Aedes aegypti* mosquito midgut extracts: Unraveling the blood meal protein digestion system,**" based on Editor and Reviewers' comments. Below is a point-by-point response to each comment.

Additional Editor Comments:

- The authors are planning to design highly selective protease inhibitors to inhibit the mosquito midgut proteases to reduce fecundity. It is not clear how such inhibitors be delivered to mosquitoes as the inhibitors have to be in human blood. The authors should add a short paragraph on how such inhibitors be used to reduce fecundity.

  The following text is included in the Conclusion/Future Directions section to address how the inhibitors can be delivered. "To deliver these inhibitors to the mosquito midgut, the attractive toxic sugar bait (ATSB) approach can be taken. ATSB exploits the need of a sugar meal by both male and female mosquitoes. Adult mosquitoes after eclosion have depleted energy stores and must replenish them either through sugar or blood feeding. By adding a toxin to the sugar bait (in this case the protease inhibitor) when the mosquitoes feed the inhibitor will end up in the midgut".

- By docking studies, the authors should also identify the residues that interact with P1 and P1' residues that define the substrate binding pockets and cleavage site selectivity.

  This is a valuable recommendation. The potential contacts for P1 and/or P1' are now indicated in the main text and added to a table in each docking figure (Figs. 8 and Fig. 9). Also, we have highlighted the measured distances between residues and P positions to better depict each interaction.

Reviewers' Comments:

Methods

- **Reviewer #1:** The reviewer only considers one major critique about the design of this study. The major concern is that the authors did not employ statistical analyses to support their conclusions? This critique covers parts of figures 2, 3 and 4.
- For Fig. 2B&C, the authors need to clearly/ define what the error bars signify (SDev, SEM ...etc)?

  In Fig. 2B and 2C the error bars signify the standard deviation of three technical replicates. We have now added this information to the Figure 2 legend.

- For Fig. 3C, are the differences in enzymatic activity between sugar and blood-fed samples significant?

  We have performed a pairwise t-test and determined that the activity in the sugar-fed samples is significantly higher ($p < 0.01$) than both sugar-fed samples after 60 minutes incubation. The blood fed samples (6h versus 24h) are not significantly different from each other. We have updated Fig. 3C with this information and added the appropriate text to the figure legend.

- For Figure 3D, the authors did not clearly/ define what the error bars signify (SDev, SEM ...etc)? Why or why not are the differences in Peak Area between 6h and 24h blood-fed significant?

  We have now indicated that the error bars in Fig. 3C and 3D correspond to the standard deviation. In Fig. 3D, the blood-fed samples have significantly higher activity using this substrate but there is no significant difference between the two blood-fed samples (6h versus 24h). We have updated Fig. 3D with this information and added the appropriate text to the figure legend.

The College of Liberal Arts and Sciences School of Molecular Sciences
PO Box 871604, Tempe, AZ 85287-1604
p: 480-965-3461 **f:** 480-965-2747 **web:** sms.asu.edu

- For Fig. 3G, If the graphs were from 4-techical replicates, why no error boars from the measure of fluorescence signal?

  The error bars are present on this graph but are too small to be visible. Below is the raw data before it was averaged. You will see that the replicate data points overlap with each other.

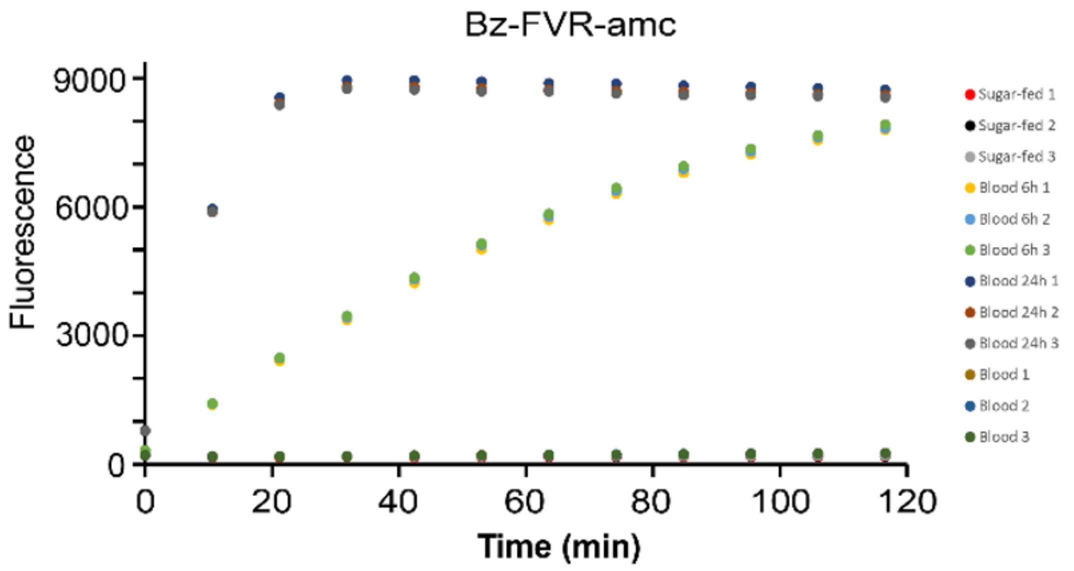

- Fig. 4 A&B, No control for AEBSF baseline activity (i.e non-fed conditions). What are the error bars? SDev, SEM ...etc. how many replicates? For which RNAi knock-downs are the differences significant?

  For Fig.4A, we did not perform a control assay using the sugar-fed midguts because in the earlier study (Fig 3G), we could not detect any protease activity in sugar-fed midgut samples using Bz-FVR-AMC. Therefore, there was no reason to test sugar-fed midguts in the presence and absence of AEBSF, since the activity of both would be zero.

  The error bars on Fig 4A and 4B is standard deviations. All assays were performed with 3 technical replicates and we have now indicated statistical significance. The figure legend has also been updated with this information.

Reviewer #2: The study by O'Donoghue et al. profiled the midgut proteolytic activity of Aedes aegypti using Multiplex Substrate Profiling by Mass Spectrometry (MSP-MS), recombinant expression and characterization of recombinant midgut proteases, and molecular docking simulations. The objectives of the study were clear (characterizing substrate specificity of Ae. aegypti midgut proteases), the methods were appropriate, and the objective impressively addressed through multiple approaches.

- However, there is detail missing in the described methodology that will need to be provided, in particular there is not much information given regarding midgut sample numbers and additional details regarding the library of designed peptides is needed. It would have good to have seen a blood only control (no mosquito midguts) with the MSP-MS.

  The design of the peptide library that is used in this study is described extensively in two previous publications by our group. 1) O'Donoghue et al Nat Methods. 2012 Nov;9(11):1095-100 and 2) Rohweder PJ et al Methods Enzymol. 2022 Dec 21;682:375–411. We have included both references in the paper.

Each sample consisted of a pool of 20 midguts and from this pool mass spectrometry-based assays were performed in 4 technical replicates while fluorescent assays were performed using 3 technical replicates. We have added this information to the manuscript.

In this study, we did not perform a blood only control MSP-MS assay. However, we have previously published a study on blood (plasma) proteases and revealed that the activity in dominated by aminopeptidases and carboxypeptidases. We have now referenced this study in our manuscript (Maffioli E et al Molecules 2020 Sep 6:25(18):4071).

Results

- **Reviewer #1:** The reviewer agrees that the figures are of sufficient quality, with the suggestion that the authors used a larger font size in the figure graphs. The reviewer suggest that the authors add the legends to Figure 3C like the ones on Figure 3D and 3G for to be consistent.

  We have modified Fig. 3 so that the font size is bigger and the legend is included for Fig. 3C.

- **Reviewer #2:** Results were clearly presented and figures of sufficient quality for publication. My only comment would be that for the molecular docking studies, it would be good to have a table that displays the distances, highlighting which distances were considered possible for nucleophile attack. The molecular docking simulation results were a bit vague.

  We appreciate this suggestion. A table was added to highlight the predicted distances for a feasible nucleophile attack among all enzymes and assessed substrates. This table can be found in Figures 8 and 9, and it also contains the P1 and/or P1' potential contacts. In addition, we further described the docking interactions in the main text, aiming to clarify the simulation results.

Conclusions

- **Reviewer #2:** Conclusions are well support, and the research question has been addressed with multiple approaches (MSP-MS, recombinant expression of proteases, and molecular docking simulations). It would be good to add a bit more to the 'Conclusions and Future Directions' that more specifically describes the next steps.

  We have modified the last paragraph in the Conclusion/Future Directions per the reviewers' suggestions.

Editorial and Data Presentation Modifications?

- **Reviewer #1:** The reviewer has one minor critique about the presentation of the Results and Discussion sections. The Results and Discussion sections were stylistically inefficient because the authors began most paragraphs by rewriting or re-integrating a method into it. Furthermore, the Discussion section re-chronicled the Results figures. The Discussion section needs to be shorter and to the point.

  We have modified the Results and Discussion sections based on the reviewers' recommendations.

- **Reviewer #2:** Minor revision. The manuscript is well written with high quality figures. There are some additional details missing from the methods that will need to be added before publication.

The College of Liberal Arts and Sciences School of Molecular Sciences
PO Box 871604, Tempe, AZ 85287-1604
p: 480-965-3461 **f:** 480-965-2747 **web:** sms.asu.edu

We have now added information regarding RNAi-mediated knockdown efficiency of protease genes at the mRNA level, citations to detailed information on the design of the peptide library, and statistical data for these and the other missing data requested above.

Summary and General Comments

- **Reviewer #1:** The reviewer has one minor critique about the presentation of the Results and Discussion sections. The Results and Discussion sections were stylistically inefficient because the authors began most paragraphs by rewriting or re-integrating a method into it. Furthermore, the Discussion section re-chronicled the Results figures. The Discussion section needs to be shorter and to the point.

  We have modified the Results and Discussion sections based on the reviewers' recommendations.

- **Reviewer #2:** O'Donoghue et al. details an impressive study profiling the substrate specificity of Ae. aegytpi midgut proteases by mass spectrometry, functional characterization of recombinant proteases, and molecular docking simulations. There is detail missing in the methods and support/justifications required for some results, please find comments below:

  Methods:

  - Line 174: "maintained on 10% sucrose until needed for experiments". How long of a period was this? Was successful knockdown confirmed with qPCR? What percentage of knockdown was achieved? Was a control used? How many mosquitoes were used?

    Immediately after dsRNA microinjection, mosquitoes were allowed to feed on 10% sucrose until dissection (please see Figure 1). 90-95% RNAi knockdown efficiency of each protease target was achieved from 12 individual mosquitoes by qPCR using firefly luciferase control dsRNA microinjection. The information has been added to the Methods section.

  - Line 179: "the tetradecapeptide library consists of 228 rationally designed peptides" Could more information be provided here? Did the authors design these peptides or was this a commercially available synthetic library?

    The design and validation of the peptide library that is used in this study is described extensively in two previous publications by our group. 1) O'Donoghue et al Nat Methods. 2012 Nov;9(11):1095-100 and 2) Rohweder PJ et al Methods Enzymol. 2022 Dec 21;682:375–411. We have included both references in the paper.

  - Line 185: How many midgut samples? Was the whole synthetic library incubated with human blood (no midgut extracts) as a control?

    There was a total of 20 midguts per each sugar-fed, 6 h and 24 h post-blood fed samples in 200 uL buffer. This information has been updated in the manuscript.

    In this study, we did not perform a blood only control MSP-MS assay. However, we have previously published a study on blood (plasma) proteases and revealed that the activity in dominated by aminopeptidases and carboxypeptidases. We have now referenced this study in our manuscript (Maffioli E et al Molecules 2020 Sep 6:25(18):4071) and added the information to the manuscript.

The College of Liberal Arts and Sciences School of Molecular Sciences
PO Box 871604, Tempe, AZ 85287-1604
p: 480-965-3461 **f:** 480-965-2747 **web:** sms.asu.edu

- I do not see where the mass spectrometry data has been made publicly available (MassIVE, Figshare, etc.). This is a requirement of PLOS Data policy.

  The raw data has been uploaded to MassIVE and a link has been provided in the Methods section.

Results

- I feel it is a bit unclear on the reasoning behind why these proteases were picked for recombinant expression. More detail should be provided regarding the abundance of these proteases in the midgut (I assume they were picked because they are highly abundant in transcriptomics or proteomics data).

  The reviewer is correct, the proteases chosen for this study were identified to be the most abundantly expressed proteases in the mosquito after acquisition of a blood meal (based on both mRNA and protein expression profiles. The information is now addressed in both the Introduction and Discussion.

- What distance is considered possible for nucleophile attack? More details needed for the criteria used. It would be nice to see a table with all these distances.

  A feasible distance for nucleophile attack would ideally be in the range of 2.5 Å or lower. We referred to this distance with Reference #40 (Torrance et al (2007) and clarified it in this new version. In addition, we added a table summarizing all nucleophile distances and their feasibility to occur. Also, additional references are provided here, which support the distances of <2.8 Å (https://doi.org/10.1101/2024.02.28.582624) and ~2.7 Å (https://doi.org/10.1002/anie.202206945).

Discussion

- Line 599: What do we know about expression and abundance of these proteases during blood feeding? That should be incorporated into the discussion.

  The proteases chosen for this study were identified to be the most abundantly expressed proteases in the mosquito after acquisition of a blood meal (based on both mRNA and protein expression profiles) and the relevance has been added to the Discussion.

We believe that all points have been addressed and modified to help strengthen the manuscript. We thank the reviewers and the editors for the helpful suggestions.

Sincerely,

Alberto A. Rascón Jr.

Alberto A. Rascón, Jr.

Associate Professor – Biochemistry

The College of Liberal Arts and Sciences School of Molecular Sciences
PO Box 871604, Tempe, AZ 85287-1604
p: 480-965-3461 **f:** 480-965-2747 **web:** sms.asu.edu
